# Robust Visual Tracking Based on Adaptive Multi-Feature Fusion Using the Tracking Reliability Criterion

**DOI:** 10.3390/s20247165

**Published:** 2020-12-14

**Authors:** Lin Zhou, Han Wang, Yong Jin, Zhentao Hu, Qian Wei, Junwei Li, Jifang Li

**Affiliations:** 1 School of Computer and Information Engineering, Henan University, Kaifeng 475004, China; zhoulin@henu.edu.cn (L.Z.); jy@henu.edu.cn (Y.J.); hzt@henu.edu.cn (Z.H.); Weiqian@vip.henu.edu.cn (Q.W.); lijunwei@henu.edu.cn (J.L.); 2 School of Electrical Engineering, North China University of Water Resources and Electric Power, Zhengzhou 450000, China; lijifang@ncwu.edu.cn

**Keywords:** visual object tracking, correlation filter, feature tracking reliability criterion, adaptive feature fusion, multiple online detectors

## Abstract

Multi-resolution feature fusion DCF (Discriminative Correlation Filter) methods have significantly advanced the object tracking performance. However, careless choice and fusion of sample features make the algorithm susceptible to interference, leading to tracking failure. Some trackers embed the re-detection module to remedy tracking failures, yet distinguishing ability and stability of the sample features are scarcely considered when training the detector, resulting in low effectiveness detection. Firstly, this paper proposes a criterion of feature tracking reliability and conduct a novel feature adaptive fusion framework. The feature tracking reliability criterion is proposed to evaluate the robustness and distinguishing ability of the sample features. Secondly, a re-detection module is proposed to further avoid tracking failures and increase the accuracy of target re-detection. The re-detection module consists of multiple SVM detectors trained by different sample features. When the tracking fails, the SVM detector trained by the most reliable sample feature will be activated to recover the target and adjust the target position. Finally, comparison experiments on OTB2015 and UAV123 databases demonstrate the accuracy and robustness of the proposed method.

## 1. Introduction

Visual single-object tracking is one of the fundamental problems in computer vision, and it involves multiple research fields such as signal processing, image processing and artificial intelligence. The task of visual object tracking is to continuously localize a target in a video sequence with given prior information such as initial location and scale of the target. Classical tracker is able to quickly and accurately localize the target only in the ideal scenario. However, some factors including deformation, occlusion and illumination variation, etc., caused by complex environment make visual object tracking challenging.

### 1.1. Related Work

In general, methods related to visual object tracking can be divided into two classes: generative methods [1,2,3] and discriminative methods [4,5,6,7,8,9,10,11,12,13]. Generative approaches often need to learn a model of target appearance and locate regions with maximum similarity to target appearance. In order to cope with various disturbances caused by complex environment, generative approaches must establish an effective appearance model, which means high computation complexity. Moreover, generative approaches usually ignore the background information of the target, making it hard to accurately separate the target from the background. To tackle this problem, discriminative adaptive tracking approaches model visual object tracking as a binary classification problem, and construct a classifier to locate target in a certain region of each frame in the video sequence.

In recent years, Discriminative Correlation Filter (DCF) has shown good performance on visual tracking benchmarks [14,15,16] and attracted extensive attention from researchers. DCF approaches perform a circular sliding window sampling operation to obtain training samples firstly, and then construct a ridge regression loss function on a set of training samples to design a filter. Moreover, by using Fast Fourier Transform (FFT), DCF maps the filter learning from spatial domain to Fourier domain and lightens the computation burden of convolution operation in spatial domain. Minimum Output Sum of Squared Error (MOSSE) [17] tracker proposed by Bolme et al. firstly employed the correlation filter in adaptive tracking and got 600 FPS tracking speed. However, other performance of MOSSE including robustness and accuracy are poor due to it using grayscale feature of images only. Boddeti et al. [18,19,20] extended the DCF framework to multi-channel feature scene, using high-dimensional features such as Histogram of Oriented Gradient(HOG) [21] and Color Names [22] to enhance accuracy and robustness of tracking. Kernelized correlation filter(KCF) [22] proposed by Henriques et al. used the HOG feature to improve both the accuracy and robustness of the tracker in the scene of motion blur and illumination variation. Danelljan et al. [8] employed the Color Names feature to enhance the robustness of tracker in situations such as deformation and occlusion induced by complex environment.

Since then, many researches on specific problems related to target tracking have been proposed, which has pushed the development of DCF methods. To relieve the negative impact caused by the scale change of target, the Scale Adaptive with Multiple Features(SAMF) [9] tracker proposed by Li et al. sampled the target with different scales and implements a multiple scale searching strategy at each frame. Danelljan et al. proposed a Discriminative Scale Space Tracking(DSST) [10] algorithm, which used a one-dimensional correlation filter independent of target displacement filter to predict the target scale change, further improving the efficiency of the scale estimation algorithm. All methods above employ cosine window technique to lighten boundary effect caused by FFT transform of periodic training samples, which makes it hard for trackers to learn background information with respective to the target, thus reducing the discrimination ability of a tracker. Danelljan et.al proposed the Spatially Regularized Discriminative Correlation Filter (SRDCF) [13] based on spatial regularization constraint, which used a predefined spatial weighting function to allocate more energy at the central region of a filter, thus enhancing the discrimination ability of the tracker.

As is known, some factors including deformation, occlusion, illumination variation and background clutter may make target appearance vary significantly, thus, deteriorating the tracking performance. To enhance the robustness of tracking, several works [23,24,25] accumulated the confidence scores from different parts of the target with part-based models and then used the parts that have high confidence scores to estimate the target’s extent. However, the most widely used strategy in tracking is to establish a robust target representation by employing robust multi-category image features. For example, Staple [26] tracker used complementary features such as HOG and color histograms to train two independent filters respectively, and then combined the estimations of the two filters to predict the target’s location. The Efficient Convolution Operators (ECO) [27] tracker efficiently integrated hand-crafted features and multi-resolution deep features, leading to superior target tracking results. Some works also investigated deep neural networks such as convolutional neural network (CNNs) [28] and Faster R-CNN [29] for object tracking. Ning et al. [30] utilized the YOLO [31] detector to generate initial object proposals. Paul et al. proposed the Siam R-CNN [32] tracker, which is an adaptation of Faster R-CNN with a Siamese architecture. The Siam R-CNN tracker re-detects a template object anywhere in an image by determining if a region proposal is the same object as a template region, and regressing the bounding box for this object. However, the thousands of extracted deep feature channels which include irrelevant and redundant descriptors are not compact, leading to deteriorating of target detection performance.

Intuitively, using multiple features to train filter can improve robustness of target tracking. However, in some special tracking scenarios such as the Unmanned Aerial Vehicle (UAV) navigation [11,12], careless choice of features will make the tracker susceptible to interference. For example, in the scenario of violently changing illumination, using color feature to train the filter may lead to extremely unstable tracking model. Therefore, it is necessary to evaluate the tracking reliability of various features on specific tracking scenario, and then establish a reasonable feature fusion strategy to select the features with high discriminate capability to train the tracking model.

In addition, using multi-category image features to train trackers also increases the computation burden of systems. Some works [13,27,33] refined the model update strategies by reducing the frequency of tracker updating or compressing the space of training samples to maintain tracking efficiency. However, these strategies, which make the tracker lose the continuous information related to the appearance change of target and produce over-fitting to the current state of the target, deteriorate the tracking performance, more seriously, lead to tracking failure. Some trackers [34,35,36] rectified the target position by conducting an accurate dynamic model of the moving target, however, non-linear characteristic of the dynamic model is hard to describe. Several other methods, such as the LCT [37] tracker, used the re-detection module to retrieve the target from tracking failures. However, there are few of methods consider the distinguishing ability and stability of the sample features when training the detector, resulting in low effectiveness detection. Therefore, making use of the continuous information to reduce the probability of tracking failure and increasing the accuracy of re-detection is also a problem which is worth to be concerned.

### 1.2. Contributions

To solve the issues mentioned above, we propose a new feature adaptive fusion method in DCF framework. Moreover, we establish a re-detection module that consists of multiple detectors based on feature tracking reliability to re-detect the target in tracking failure scene, enhancing the robustness of tracking. The key innovations of the proposed method are listed as follows, 

• We propose a criterion of feature tracking reliability and conduct a novel feature adaptive fusion method in filter learning. Different from traditional multi-resolution feature fusion trackers, the proposed method can adaptively assign greater weights to those features with high reliability and background distinguishing ability and vice versa, leading to a robust and accurate tracking.

• A re-detection module consisting of multiple SVM detectors trained by samples under different image feature maps is embedded to deal with tracking failures. Each detector in the module is labeled with the tracking reliability of its sample feature. The detector with maximum label in re-detector module would be activated to locate the lost target in the scene of tracking failures.

### 1.3. Paper Organization and Notation

The rest of this paper is organized as follows. Section 2 states the traditional multi-resolution feature fusion DCF framework. Section 3 introduces the proposed method in this paper. In the Section 3.1, we define the concept and evaluation method of feature tracking reliability criterion, and state the feature adaptive fusion scheme in filter learning formulation based on the feature tracking reliability. The re-detection module with multiple online detectors is described in Section 3.2. Section 4 provides an overview of the tracking algorithm. Finally, in Section 5 and Section 6, we do experiments on OTB 2015 and UAV123 datasets to compare the proposed algorithm with competing methods in details and conclude the paper.

In this paper, we use R to denote the set of real numbers, C to denote the set of complex numbers and Z to denote the set of integers. We use capital bold case, e.g., A to denote matrices. The direct sum and Kronecker product of matrices are denoted by ⊕ and ⊗, respectively. We use bold lower case, e.g., x to denote multi-channel signal and the *d*th channel of signal x is indicated as upper case, e.g., $x^{d}$. We use $x^{T}$ and $x^{H}$ to denote the transposition and Hermitian transposition of signal x, respectively. The $L^{2}\left(T\right)$ is considered as the Hilbert space equipped with the inner product $\langle g(t),h(t)\rangle =\frac{1}{T}\int_{0}^{T}g\left(t-\tau \right)h\left(\tau \right)d\tau$. In $L^{2}\left(T\right)$, the circular convolution operation is defined as $g\left(t\right)*h\left(t\right)=\frac{1}{T}\int_{0}^{T}g\left(t-s\right)h\left(s\right)ds$. For function $g\in L^{2}\left(t\right)$, we define its squared $L^{2}$-norm as ${∥g∥}_{L^{2}}^{2}=\frac{1}{T}\int_{0}^{T}{\left|g\left(t\right)\right|}^{2}dt$ and the Fourier coefficients of *g* is indicated as $\hat{g}\left[k\right]=\frac{1}{T}\int_{0}^{T}g\left(t\right)e^{-i\frac{2\pi }{T}kt}dt,k\in Z$. The squared $\ell^{2}$-norm of $\hat{g}$ is defined as ${∥\hat{g}∥}_{\ell^{2}}^{2}=\sum_{-\infty }^{\infty }{\left|\hat{g}\left[k\right]\right|}^{2}$.

## 2. Traditional Multi-Resolution Features Fusion Filter

**Figure 1:**
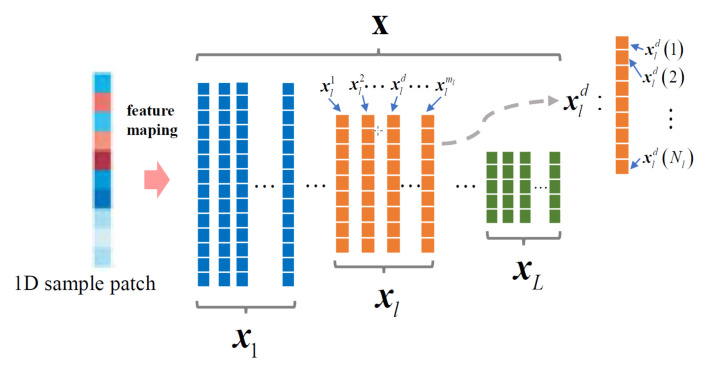
Visualization of the multi-resolution feature mapping in 1D domain.

In filter training, the aim is to obtain a convolution filter f based on a set of training samples ${\left\{\left(x_{j},y_{j}\right)\right\}}_{j=1}^{M}$ collected from frame 1 to *M*. Here, $y_{j}$ is the label of training sample $x_{j}$. For the sake of clarity, the filter learning framework in this paper is formulated on data defined in one-dimensional domain, which is displayed in Figure 1. The training sample $x=\left(x_{1},...,x_{L}\right)$ contains *L* number of feature layers with different resolutions which are extracted from the same image patch. The *l*th feature layer $x_{l}$ contains $m_{l}$ channels, namely $x_{l}=\left[x_{l}^{1},x_{l}^{2},\cdots ,x_{l}^{m_{l}}\right]$, and thus there are $D=\sum_{l=1}^{L}m_{l}$ feature channels in sample x. We use $N_{l}$ to denote the number of spatial sample points in the $x_{l}^{d}$ channel of feature layer $x_{l}$, which means $x_{l}^{d}=\left[x_{l}^{d}\left(1\right),x_{l}^{d}\left(2\right),\cdots ,x_{l}^{d}\left(N_{l}\right)\right]$. Thus, the sample space is expressed as $Ø=R^{m_{1}\times N_{1}}\times ...\times R^{m_{L}\times N_{L}}$.

To achieve sub-pixel level localization accuracy, we convert the learning problem from the discrete spatial domain to continuous spatial domain using an interpolation operator $J_{l}:R^{N_{l}}\to L^{2}\left(T\right)$, T∈R. Specifically, for each feature channel $x_{l}^{d}\in R^{N_{l}}$, in feature layer $x_{l}$, the interpolation operator is described as

(1) $$J_{l}\left\{x_{l}^{d}\right\}\left(t\right)=\sum_{n=0}^{N_{l}-1}x_{l}^{d}\left[n\right]b\left(t-\frac{T}{N_{l}}n\right),d=1,...,m_{l}$$

where $x_{l}^{d}\in R^{N_{l}},\;\;\;d=1,...,m_{l}$, and *T* represents the size of the support region. $J_{l}\left\{x_{l}^{d}\right\}$ is thus a continuous *T*-periodic real function. The interpolation function $b_{l}$ is constructed using the standard cubic spline kernel [27],

(2) $$b\left(t\right)=\left\{\begin{array}{cc} \left(a+2\right){\left|t\right|}^{3}-\left(a+3\right)t^{2}+1 & \left|t\right|⩽1\\ a{\left|t\right|}^{3}-5at^{2}+8a\left|t\right|-4a & 1\leq \left|t\right|<2\\ 0 & \left|t\right|>2 \end{array}\right.$$

where *a* is the shape parameter of *b*. Simultaneously, the label function is also converted into continuous spatial domain, which means $y_{j}\in L^{2}\left(T\right)$.

In the traditional filter learning framework, the objective function of the filter f is described as,

(3) $$\min E\left(f\right)=\sum_{j=1}^{M}\alpha_{j}{∥\sum_{l=1}^{L}\sum_{d=1}^{m_{l}}f_{l}^{d}*J_{l}\left\{x_{l}^{d}\right\}-y_{j}∥}_{L^{2}}^{2}+\sum_{l=1}^{L}\sum_{d=1}^{m_{l}}{∥wf_{l}^{d}∥}_{L^{2}}^{2}$$

by solving the Equation (3), we can obtain a multi-channel convolution operator $S_{f}$ which is parameterized by a bank of convolution filters $f=\left(f_{1},...,f_{L}\right)$, $f_{l}=\left(f_{l}^{1},...,f_{l}^{m_{l}}\right)$. The operator $S_{f}$ maps a sample x into a target confidence function that can be described as

(4) $$S_{f}\left\{x\right\}\left(t\right)=f*J\left\{x\right\}=\sum_{l=1}^{L}\sum_{d=1}^{m_{l}}f_{l}^{d}*J_{l}\left\{x_{l}^{d}\right\},x\in Ø$$

The $S_{f}\left\{x\right\}\left(t\right)$ represents the confidence score of the target at the location $t\in \left[0,T\right)$ of the image. Similar to other discriminative methods, the target is localized at the position with the maximum confidence score in the image region.

It can be obviously seen in Equation (3) that there are *L* kinds of multi-resolution features employed in filter training to enhance the robustness of the tracker. However, the weights of these features are constant during the whole tracking process. As mentioned in Section 1, the rough feature fusion strategy makes the tracker susceptible to the interference in the scene of features changing violently.

## 3. The Proposed Method

To tackle the problems mentioned above, we propose a novel feature adaptive fusion strategy into tracker training framework in Section 3.1. The feature adaptive fusion strategy is formulated based on our criterion of feature tracking reliability, which is defined to evaluate the distinguishing ability and robustness of the features. In Section 3.2, we propose a re-detection module consisting of multiple SVM detectors trained by different sample features to deal with tracking failures. The re-detection module can further avoid the tracker losing the continuous information of the target appearance and producing over-fitting to the current state of the target.

### 3.1. Feature Adaptive Fusion Strategy in Filter Training

To tackle the problem mentioned at the end of the Section 2, this paper proposes a novel feature adaptive fusion strategy into filter training objective function as,

(5) $$\min E\left(f\right)=\sum_{j=1}^{M}\alpha_{j}{∥\sum_{l=1}^{L}\sum_{d=1}^{m_{l}}f_{l}^{d}*r_{l}J_{l}\left\{x_{l}^{d}\right\}-y_{j}∥}_{L^{2}}^{2}+\sum_{l=1}^{L}\sum_{d=1}^{m_{l}}{∥wf_{l}^{d}∥}_{L^{2}}^{2}$$

In Equation (5) each kind of feature $x_{1}$ is weighted by its feature tracking reliability $r_{l}\in R$, which is different from the traditional objective function described in Equation (3). The feature weight $r_{l}$ is calculated by our “feature tracking reliability criterion” which is defined as,

(6) $$r_{l}=\zeta \max\left(S_{f}^{l}\left\{x\right\}\right)=\zeta \max\left(\sum_{d=1}^{m_{l}}f_{l}^{d}*J_{l}\left\{x_{l}^{d}\right\}\right)$$

where the $S_{f}^{l}\left\{x\right\}=\sum_{d=1}^{m_{l}}f_{l}^{d}*J_{l}\left\{x_{l}^{d}\right\}$ is the filter output of the feature layer $x_{1}$. The normalization scalar ζ ensures that $\sum_{l=1}^{L}r_{l}=1$.

The feature tracking reliability criterion defined in Equation (6) is formulated based on the fact that the Equation (3) independently solves the least squares problem over all feature layers. Thus, the output of each feature layer $S_{f}^{l}\left\{x\right\}$ should nearly exactly fit the ideal response y. On the other hand, the response is highly noisy on the feature layers with low discriminative power. The existing noise leads to significant global error reduction of least squares, moreover, reduces the maximal output of the response related to the feature layers [38]. Therefore, robustness and background distinguishing ability of the *l*th sample feature can be evaluated by the maximum output response of $x_{1}$. Thus, according to the feature tracking reliability criterion defined in Equation (6), the proposed method can adaptively assign greater weights to those features with high reliability and background distinction. The features in Equation (5) are suitably fused to enhance the robustness of our tracking model.

In practice, the filter in Equation (5) can be efficiently solved by using FFT transform. However, in order to further lighten the computation burden, we dispose the features with dimensionality reduction technique. For feature layer $x_{l}$, suppose there is a $m_{l}\times c_{l},\left(m_{l}>c_{l}\right)$ matrix $P_{l}=\left\{p_{d,c}\right\},d=1,...,m_{l};c=1,...,c_{l}$, which makes $S_{f}^{l}\left\{x\right\}=S_{Pf}^{l}\left\{x\right\}=P_{l}f_{l}*J_{l}\left\{x_{l}\right\}=\sum_{d,c}p_{d,c}f_{l}^{c}*J_{l}\left\{x_{l}^{d}\right\}\;=\;f_{l}*P_{l}^{T}J_{l}\left\{x_{l}\right\}$, i.e., each filter $f_{l}^{1},...,f_{l}^{m_{l}}$ can be linearly combined by a smaller set of basis filters $f_{l}^{1},...,f_{l}^{c_{l}}$. The filter learning objective function in Fourier domain is finally derived as

(7) $$\min E\left(P,f\right)=\sum_{j=1}^{M}\alpha_{j}{∥\sum_{l=1}^{L}r_{l}{\hat{z}}_{l}^{T}P_{l}{\hat{f}}_{l}-{\hat{y}}_{j}∥}_{\ell^{2}}^{2}+\sum_{l=1}^{L}\sum_{c=1}^{c_{l}}{∥\hat{w}*{\hat{f}}_{l}^{c}∥}_{\ell^{2}}^{2}+\sum_{l=1}^{L}\lambda {∥P_{l}∥}_{F}^{2}$$

here, we use ${\hat{z}}_{l}\left[k\right]=X_{l}^{d}\left[k\right]\hat{b_{l}}\left[k\right]$ to denote the Fourier coefficients related to the feature map $z_{l}=J_{l}\left\{x_{l}\right\}$ in the *l*th feature layer. The $X_{l}^{d}=\sum_{n=0}^{N_{l}}x_{l}^{d}\left[n\right]e^{-i\frac{2\pi }{N_{l}}nk},k\in Z$ is the Discrete Fourier Transform (DFT) of $x_{l}^{d}\left[n\right]$. It should be noted that the matrix $P_{l}$ is initialized on the first frame by operating the principal component analysis (PCA) on the feature $x_{l}$. We update the $P_{l}$ according the sample features extracted in current frame. To ensure the stability of the target model, the Frobenius norm of matrix $P_{l}$ controlled by the weight parameter λ is added as a regular term to limit the updating of $P_{l}$.

In conclusion, according to the feature tracking reliability criterion defined in Equation (6), our method can adaptively fuse the sample features used in filter training in Equation (5). With the FFT transform and dimensionality reduction technique, the Equation (5) can be transformed into Equation (7), and the filter can be efficiently learned and updated in Fourier domain by solving Equation (7) using Gauss-Newton and Conjugate Gradient method. In the target detection stage, the output response of the tracker thus can be calculated as

(8) $$S_{f}\left\{x\right\}=\sum_{l=1}^{L}r_{l}S_{f}^{l}\left\{x\right\}=\sum_{l=1}^{L}r_{l}f_{l}*P_{l}^{T}J_{l}\left\{x_{l}\right\},x\in Ø$$

and the target is localized at the position with the maximum confidence score in the image region.

### 3.2. Multiple Online Detectors Based on Feature Tracking Reliability

Employing a Gaussian Mixture Model [27], we manage the training samples collected in each frame to construct a compact sample space with $N_{s}$ training samples. This operation can eliminate the redundant information among the training samples. In addition, we update the filter once every $N_{u}$th frame to further improve tracking efficiency. However, as mentioned in Section 1, above strategies may bring about some continuous information loss related to the appearance change of target.

To tackle this problem, we additionally train a long-term filter $S_{L}\left\{x\right\}$ with DCF framework to record and track the appearance change of the target firstly. The maximum response of the long-term filter $A_{L}=\max S_{L}\left\{x\right\}$, which is obtained at the estimated position in each frame is to be solved to detect tracking failure. In the case of $A_{L}=\max S_{L}\left\{x\right\}<T_{r}$ , where the $T_{r}$ is a constant threshold, it is believed that there is a tracking failure, then a detection module will be activated to re-detect the target and revise the tracking result.

Different from traditional detection methods, this paper builds multiple SVM detectors $\left[h_{1},...,h_{L}\right]$ using training samples under different image feature maps independently. Each detector $h_{l}$ is labeled with the feature tracking reliability $r_{l}$ related to the feature map of its train samples. When the tracking fails, we use the SVM detector with the maximum label to redetect the target.

Given a training set $\left\{\left(v_{i},b_{i}\right),i=1,...,N,b_{i}\in \left\{+1,-1\right\}\right\}$ in a frame, The objective function related to multiple SVM detectors is

(9) $$\min_{h}\frac{1}{2}{∥h∥}^{2}+\frac{1}{N}\sum_{i}L\left(h;\left(v_{i},b_{i}\right)\right)$$

where the loss function can be defined as $L\left(h;\left(v,b\right)\right)=\max\left\{0,1-b\langle h,v\rangle \right\}$. We can efficiently update h by

(10) $$h\leftarrow h-\frac{L\left(h;\left(v,b\right)\right)}{{∥\nabla_{h}L\left(h;\left(v,b\right)\right)∥}^{2}+\frac{1}{2\tau }}\nabla_{h}L\left(h;\left(v,b\right)\right)$$

here, the $\nabla_{h}L\left(h;\left(v,b\right)\right)$ is the gradient of the loss function in terms of h and the $\tau \in \left(0,+\infty \right)$ is a hyper-parameter that controls the update rate of h. It should be noted that in Equations (9) and (10), the training sample $v=\left[v_{1},...,v_{L}\right]$ consists of *L* number of feature layers extracted from the sample patch. The $h=\left(h_{1},...,h_{L}\right)$ denotes a hyperplane set with respect to *L* number of SVM detectors. Each SVM detector $h_{l}$ in h is labeled by the feature tracking reliability $r_{l}$ of its sample feature. Only the detector with the maximum label will be activated to redetect the target when tracking failure occurs.

## 4. Outline of the Proposed Method

**Figure 2:**
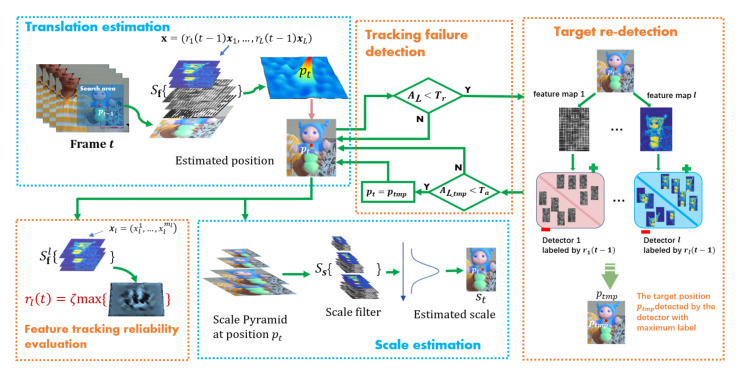
Overview of the proposed algorithm.

This paper presents an overview of the proposed method in Figure 2. It can be seen that the whole tracking process of the proposed method can be divided into five parts, i.e., the Translation estimation part which is described in Equation (9) in Section 3.1, the Tracking failure detection and Target re-detection parts described in Section 3.2, the Feature tracking reliability evaluation part described in Equation (6) and the Scale estimation part. It should be noted that in the Scale estimation part, we design an 1-dimensional scale filter $S_{s}$ to estimate the scale of target using the same way as DSST [10]. We also design multi-resolution feature maps related to samples for filter $S_{s}$ and assign weights of these features according to their feature tracking reliability $r_{l}$. More details of the tracking process in Figure 2 are discussed in the following Table 1.

In Figure 2 and Table 1, the constant $T_{r}$ represents the threshold of starting the detection module, and the constant $T_{a}$ is the threshold of adopting the re-detection result. Meanwhile, the stability threshold $T_{s}$ in Table 1 is introduced to determine the filter $S_{L}$ and detectors updating. Specifically, $S_{L}$ and multiple SVM detectors are updated only when the maximum response of $S_{L}\left\{x\right\}$ is greater than $T_{s}$. Some same resolution features including gradient-based features (HOG) and intensity-based features (HOI) are employed to train Long term filter. The feature tracking reliability $r_{l}$ and filters $S_{f}$, $S_{s}$ and $S_{L}$ are updated by a moving average scheme.

When updating the filter $S_{f}$ using Equation (7), we independently employ the Gauss-Newton method on each feature layer. For simplicity, we consider learning the filter $f_{l}$ of the feature layer $x_{l}$ from a single training sample x. The corresponding loss function in Equation (7) is derived as

(11) $$\min E\left(P{}_{l}f_{l}\right)={∥r_{l}{\hat{z}}_{l}^{T}P_{l}{\hat{f}}_{l}-{\hat{y}}_{j}∥}_{\ell^{2}}^{2}+\sum_{c=1}^{c_{l}}{∥\hat{w}*{\hat{f}}_{l}^{c}∥}_{\ell^{2}}^{2}+\lambda {∥P_{l}∥}_{F}^{2}$$

The Gauss-Newton method is derived by linearizing the residuals in Equation (11) using a first order Taylor series expansion, which approximates the bi-linear term ${\hat{z}}_{l}^{T}P_{l}\hat{f_{l}}$ around the current estimate $\left({\hat{f}}_{l\left(i\right)},P_{l\left(i\right)}\right)$ at iteration *i* as

(12) $$\begin{array}{cc} \; & {\hat{z}}_{l}^{T}\left(P_{l\left(i\right)}+\Delta P_{l}\right)\left({\hat{f}}_{l\left(i\right)}+\Delta {\hat{f}}_{l}\right)\\ \; & \approx {\hat{z}}_{l}^{T}P_{l\left(i\right)}{\hat{f}}_{l\left(i\right),\Delta }+{\hat{z}}_{l}^{T}\Delta P_{l}{\hat{f}}_{l\left(i\right)}\;=\;{\hat{z}}_{l}^{T}P_{l\left(i\right)}{\hat{f}}_{l\left(i\right),\Delta }\;+\;{\left({\hat{f}}_{l\left(i\right)}⊗{\hat{z}}_{l}\right)}^{T}\mathrm{vec}\left(\Delta P_{l}\right) \end{array}$$

here, the “vec” represents the vectorization operation of the matrix, we define ${\hat{f}}_{l\left(i\right),\Delta }\;=\;{\hat{f}}_{l\left(i\right)}+\Delta \hat{f_{l}}$, the Gauss-Newton subproblem at iteration is derived by substituting the first-order approximation Equation (12) into Equation (11)

(13) $$\begin{array}{cc} \; & \min E\left(\Delta P_{l},{\hat{f}}_{l\left(i\right),\Delta }\right)\\ & ={∥r_{l}\left({\hat{z_{l}}}^{T}P_{l\left(i\right)}{\hat{f}}_{l\left(i\right),\Delta }\;+\;{\left({\hat{f}}_{l\left(i\right)}⊗{\hat{z}}_{l}\right)}^{T}\mathrm{vec}\left(\Delta P_{l}\right)\right)-{\hat{y}}_{j}∥}_{\ell^{2}}^{2}\\ \; & \;+\;\sum_{c=1}^{c_{l}}{∥\hat{w}*{\hat{f}}_{{}_{l\left(i\right),\Delta }}^{c}∥}_{\ell^{2}}^{2}+\lambda {∥P_{l\left(i\right)}+\Delta P_{l}∥}_{F}^{2} \end{array}$$

Since the filter $f_{l}$ is constrained to have finite non-zero Fourier coefficients, Equation (13) is a linear least squares problem. We employ the Conjugate Gradient method to optimize the Gauss-Newton subproblem at each iteration.

## 5. Experiments and Analysis

Performances related to the proposed method are verified by comparison experiments on OTB2015 (http://cvlab.hanyang.ac.kr/tracker_benchmark/datasets.html) and UAV123 (https://cemse.kaust.edu.sa/ivul/uav123) datasets. As we know, OTB2015 [15] dataset is the most popular tracking benchmark with 100 video sequences which are fully annotated with 11 different attributes. The UAV123 [16] dataset contains a total of 123 video sequences from an aerial viewpoint. To demonstrate the performance of proposed tracker, some state-of-the-art trackers, including ECO [27], CSR_DCF [27], SRDCF [13], Staple [26], DSST [10] and LCT [34] are used to compare with our tracker. It should be noted that only single-object tracking task is considered in the comparison of this paper. For a fair comparison, the performances of the 7 trackers above are compared under the same environment conditions using MATLAB2016b equipped with Windows 10-64bit on Intel(R) Core (TM) i5-9300H CPU and 8GB RAM.

### 5.1. Experimental Parameters

The proposed method uses two hand-crafted image feature layers, namely Color Names which reflects the color information of the target and HOG layer which reflects the spatial structure and texture characteristics of the target, to demonstrate the performance of the proposed method. The experimental parameters are described in Table 2. The parameters related to the long term filter $S_{L}$ and the scale filter $S_{s}$ are selected referring to the LCT [34] and DSST [10] trackers. The parameters in Table 2 are chosen based on the ECO [27] and CSR_DCF [35] trackers and fine-tuned according to the tracking AUC performance.

### 5.2. Evaluation Indicators

This paper uses One Pass Evaluation (OPE) criterion including center location error and the bounding box overlap score to evaluate the performance of trackers. We also employ the Success plot, Mean Distance Precision (Mean DP), Mean Overlap Precision (Mean OP), average center location error (CLE) and area-under-curve (AUC) as the expressions of the experiment results. It should be noted that given a estimated bounding box ${ROI}_{e}$ and the ground-truth bounding box ${ROI}_{g}$ of the target, the bounding box overlap score is defined as

(14) $$IOU=\frac{area\left({ROI}_{e}\cap {ROI}_{g}\right)}{area\left({ROI}_{e}\cup {ROI}_{g}\right)}$$

Since involving the position and scale related to tracked target simultaneously, IOU is an indicator different with the center location error, and it can widely be used to evaluate the robustness and accuracy of the tracking algorithm.

### 5.3. Comparisons and Analysis

#### 5.3.1. Impact of the Feature Adaptive Fusion

**Figure 3:**
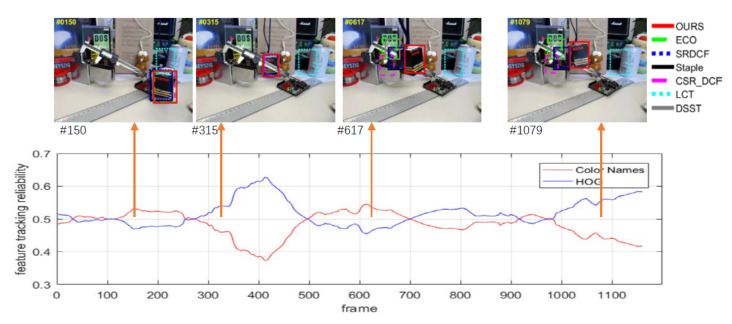
Visualization of the feature weights change in adaptive feature fusion.

In the section, we firstly evaluate the effect of feature adaptive fusion based on the feature tracking reliability criterion in our method. The proposed method can adaptively evaluate the tracking reliability of each feature in the target model according to the Equation (6). The tracking reliability of each feature is then used in Equations (5) and (7) as its feature adaptive fusion weight in constructing the target model. Thus, our method can adaptively increase the weights of feature with more stability, robustness and distinction while reduce the weights of the features with poor stability, thereby leading to a robust and accurate tracking. To evaluate the effect of the feature adaptive fusion proposed in this paper, we demonstrate the tracking process on video sequence named “Box” in Figure 3. We present the tracking bounding box comparison of the proposed method with the comparing trackers at the top of the Figure 3, and the change of sample feature tracking reliability calculated using Equation (6) in our method is illustrated at the bottom of the Figure 3.

As shown in Figure 3, in the 151st frame, due to the non-planar rotation of the target, the HOG feature of the target fluctuates greatly, leading to locating failure of the target, while the color information of the target is still stable. The proposed algorithm can adaptively increase the feature weight of the Color Names and stably track the target; In the 315th frame, the target is partially occluded, the feature is highly noisy in the color information of the target. The algorithm can adaptively increase the weight of the HOG feature and use the structure information to track the target stably; In the 617th frame, when the target appears again from the full occlusion, although the rectangular structure of the target has changed greatly due to the rotation, the target can still be detected by the detector of our method trained with the relatively stable color features; After the 1000th frame, the target’s spatial structure tends to be stable, the algorithm thus adaptively increases the weight of the HOG feature to track the target.

#### 5.3.2. Baseline Comparison

In this section, we report the performances of the mean overlap precision (Mean OP), mean distance precision (Mean DP), center location error (CLE) and Success plot related to all the methods on OTB 2015 and UAV123 datasets. It should be noted that the overlap precision score (OP) is defined as the ratio of frames in a video which the IOU is greater than a certain threshold op(op=0.5). The distance precision score (DP) is defined as the ratio of frames in a video where the Euclidean distance between the tracking output and ground truth is smaller than a threshold dp(dp=20(pixel)).

**Figure 4:**
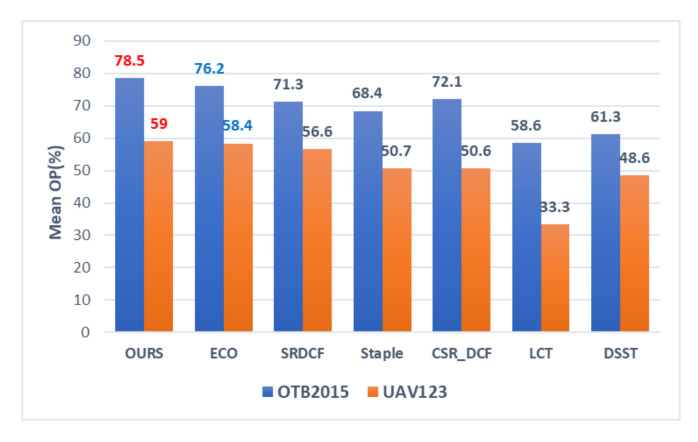
Comparison of Mean overlap precision score (OP) on OTB 2015 and UAV123 datasets.

**Figure 5:**
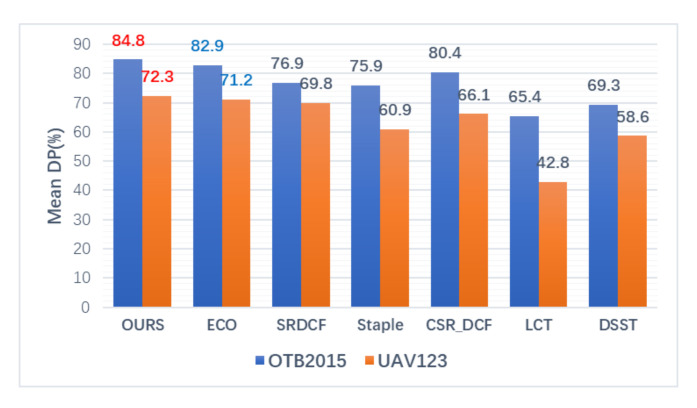
Comparison of Mean distance precision score (DP) on OTB 2015 and UAV123 datasets.

**Figure 6:**
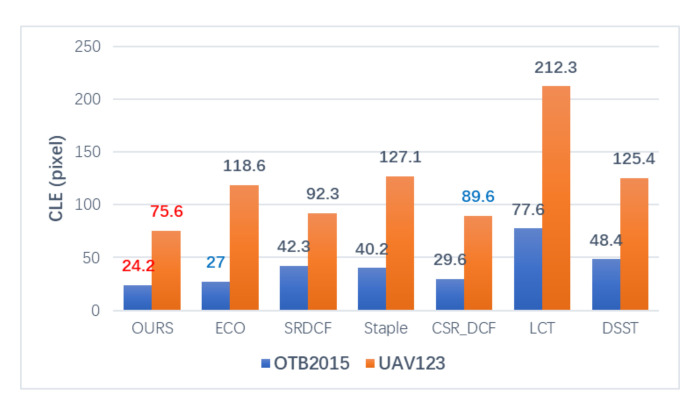
Comparison of center location error (CLE) on OTB 2015 and UAV123 datasets.

We present the Mean OP, Mean DP and CLE comparisons of the trackers respectively in the histograms of Figure 4, Figure 5 and Figure 6, and the best two results are highlighted in red and blue fonts. It can be seen that the proposed method gets the highest Mean OP in Figure 4 and the highest Mean DP in Figure 5 on both OTB2015 and UAV123 datasets. In Figure 6, our method gets the lowest CLE scores. Compared with the second-best method, we can find that our method achieves a gain of 2.3% in Figure 4 of the Mean OP, 1.9% in Figure 5 of the Mean DP, and 2.78 pixels in Figure 6 of the CLE on OTB2015 dataset and a gain of 0.6% in Figure 4 of the Mean OP, 1.1% in Figure 5 of the Mean DP, and 43 pixels in Figure 6 of the CLE on the UAV123 dataset, respectively.

**Figure 7:**
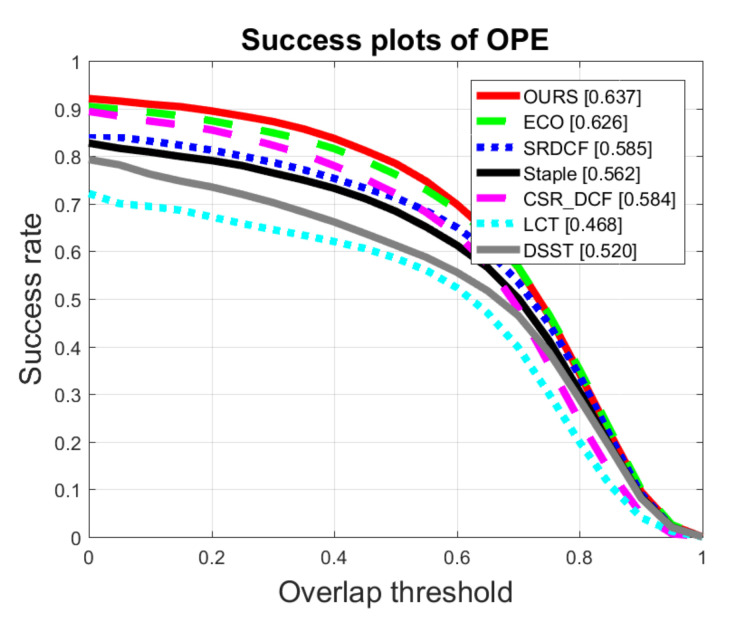
Comparison of Success plots with 7 trackers on OTB2015.

**Figure 8:**
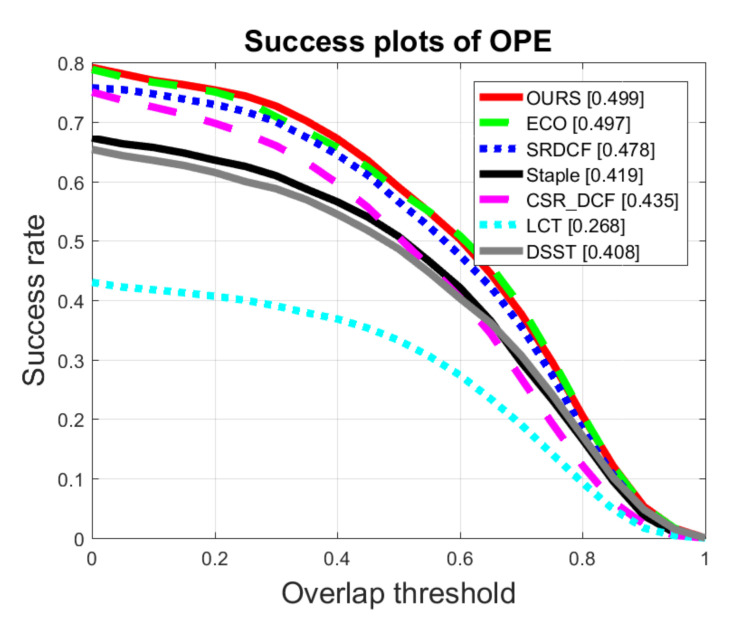
Comparison of Success plots with 7 trackers on UAV123.

The Success plots and the AUC scores of all the trackers on OTB2015 and UAV123 datasets are shown in Figure 7 and Figure 8, respectively. On OTB2015 dataset illustrated in Figure 7, the ECO tracker achieves an AUC score of 62.6%, while our method achieves an AUC score of 63.7%, 1.1% higher than that of the second-best ECO tracker. Meanwhile, on UAV123 dataset illustrated in Figure 8, our method obtains an AUC score of 49.9%, 0.2% higher than that of the second-best method.

Since considering feature tracking reliability in filter learning, our method can adaptively select features with high discrimination for tracking model training. Moreover, a re-detection module is introduced in tracking process to re-detect the target and to align the estimated position in the case of tracking failure. So, our method shows the best performance on the OTB 2015 and UAV123 datasets with the metrics including Mean OP, Mean DP, CLE and Success plot.

#### 5.3.3. Attribute-Based Comparison

**Figure 9:**
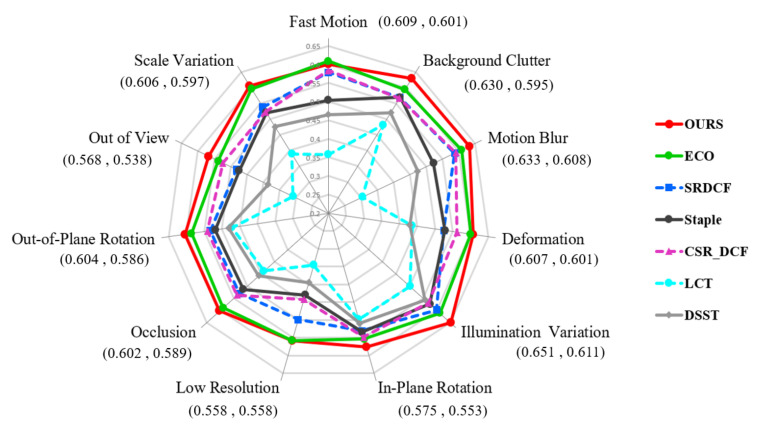
Comparison of area-under-curve (AUC) scores on all visual attributes on OTB2015.

We further perform an attribute-based analysis of all the methods respectively on the OTB 2015 and UAV 123 datasets. In OTB2015 dataset, all the sequences are annotated with 11 different attributes. As shown on the 11 attribute axes in Figure 9, the 11 video attributes are: Fast Motion, Background Clutter, Motion Blur, Deformation, Illumination Variation, In-Plane Rotation, Low Resolution, Occlusion, Out-of-Plane Rotation, Out of View and Scale Variation.

**Figure 10:**
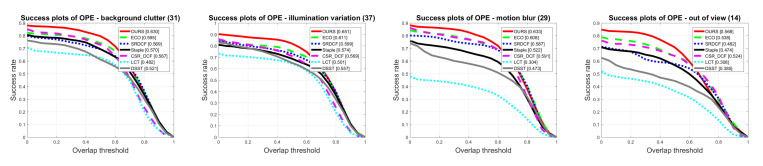
Success plots about Background Clutter Illumination Variation, Motion Blur and Out of View attributes on OTB2015.

Figure 9 shows the AUC score comparisons of the proposed method with other trackers on all 11 attributes in OTB 2015. On each visual attribute axe, the AUC scores of trackers are arranged from the center of the figure to the edge in order from small to large. The AUC scores of the best two trackers are displayed behind the axe labels. It can be seen from the Figure 9 that the proposed method has superior AUC scores on the above 11 attributes and outperforms the other 6 trackers on 10 attributes except the Fast Motion. The advantages of our method are clearly embodied in four attribute axes, namely Background Clutter, Illumination Variation, Motion Blur and Out of View. The Success plots on these four attributes are illustrated in Figure 10.

In the Background Clutter case of Figure 10, the background nearby the target has similar color or texture to the target, greatly interfering with the object tracking. The proposed method can adaptively assign greater weights to features which have better distinguish capacity according to feature tracking reliability criterion, thus achieving a robust tracking. Our method achieves an AUC score of 63%, 3.5% higher than that of the second-best ECO algorithm. In the Illumination Variation case where the lighting condition changes violently, the features related to color characteristics of the target are extremely unstable and unsuitable to be used for target locating. In this case, our method can adaptively assign small weights to Color Names features that reflect the color information of the target and set greater weights to the HOG features that reflect the spatial structure and texture of the target. Thus, it can reduce damaging effect caused by illumination variation on target tracking. The AUC score of our tracker on Illumination Variation attribute reaches 65.5%, 4% higher than that of second-best ECO tracker. In Motion Blur scenario of Figure 10, the spatial structure and texture of the target tend to be unstable. However, color features can still reflect the target position. Therefore, the proposed method adaptively increases the weight of Color Names and achieves a better tracking performance. It can be seen that our method obtains an AUC score of 63.3%, 2.5% higher than that of the ECO tracker. On the Out of View attribute, the tracking process tends to fail because of temporarily disappearing of the target in the field of view. A re-detection module is introduced to detect the target position and re-initialize the tracker . The re-detection module consists of multiple SVM detectors trained by samples under different image feature maps, and the detector trained by the most reliable sample feature is activated to detect the target when the target returns to the field of view so that the tracker can continue to track the target. The AUC score of our method reaches 56.8%, 3% higher than that of the second-best method.

**Figure 11:**
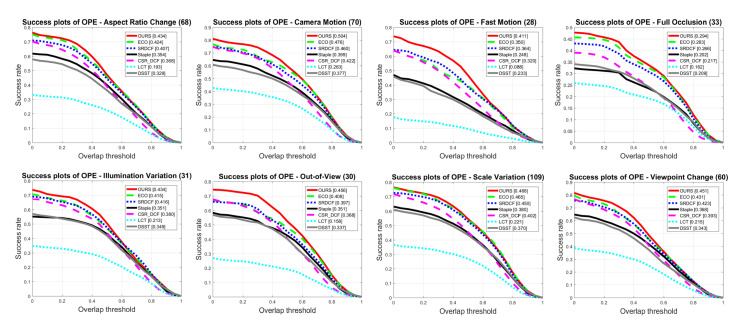
Success plots of trackers with 8 attributes on the UAV123 dataset.

The UAV123 dataset consists of 12 different attributes, namely Illumination Variation (IV), Scale Variation (SV), Partial Occlusion (POC), Full Occlusion (FOC), Out-of-View (OV), Fast Motion (FM), Camera Motion (CM), Background Clutter (BC), Similar Object (SOB), Aspect Ratio Change (ARC), Viewpoint Change (VC) and Low Resolution (LR). The proposed tracker in this paper performs favorably against other trackers in most attributes defined in the UAV123 dataset. Examples of success plots are demonstrated in Figure 11. It can be clearly seen that the curve corresponding to our tracking method is always higher than that corresponding to other trackers on the 8 Success plots in Figure 11, indicating that the proposed method outperforms the other 6 trackers on these 8 attributes. Especially, due to the adaptively fused robust multi-resolution feature representation and carefully choice of SVM detectors, our method greatly improved the tracking performance in videos with Camera Motion, Fast Motion and Out-of-View attributes. It can be seen that our method gets AUC sco res of 50.4%, 41.1% and 45.6% respectively on Fast Motion and Out-of-View attributes, which are 2.8%, 6.1% and 4.8% higher than the second-best tracker.

**Figure 12:**
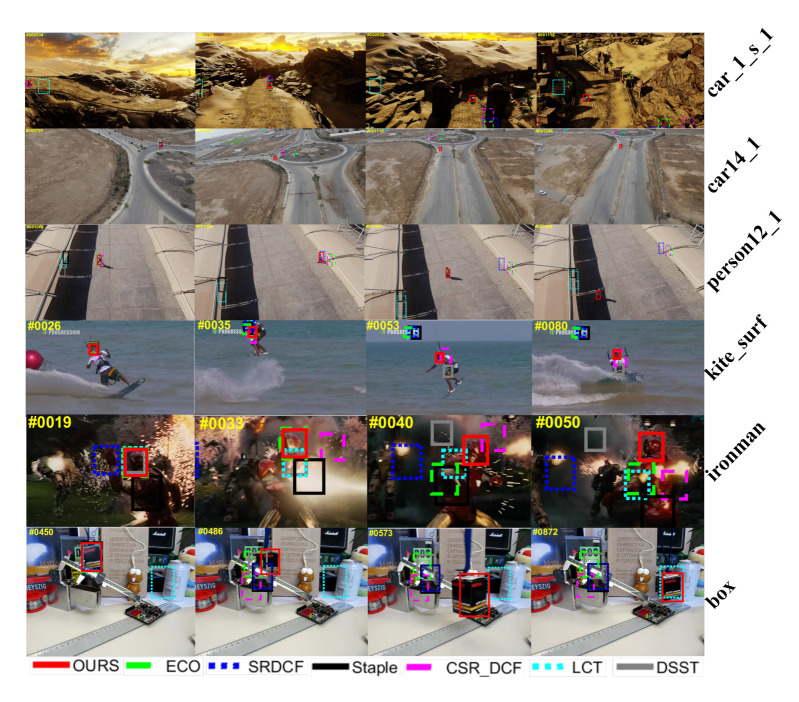
Qualitative comparison of the trackers on several videos.

In order to compare the tracking performance of the trackers more intuitively, a qualitative comparison of the tracking results on some video sequences from OTB2015 and UAV123 dataset is shown in Figure 12. From the comparison results on “car_1_s_1” and “ironman” sequences, we can find that the proposed tracking method is robust to the illumination variation. The comparison results of “kite_surf” and “box” sequences indicate that our method can efficiently recover the object from occlusion. It can also be proven that the tracker proposed in this paper is competent in dealing with rotation, deformation and other attributes defined in OTB2015 and UAV123 benchmarks as well. Videos demonstrating the results of the propoosed method can also be found from the link: https://drive.google.com/drive/folders/15rcmiSTqQxGFnf9Fm79d4bMX6fobkWbY?usp=sharing (You can also contact us to get the videos for free: wanghan_henu@163.com).

## 6. Conclusions

This paper firstly proposes a novel feature fusion formulation in filter learning using the criterion of feature tracking reliability. The feature tracking reliability criterion is defined to evaluate the robustness and the background distinguishing ability of the sample features. Then, a re-detection module with multiple SVM detectors labeled with the feature tracking reliability is proposed to reduce the possibility of tracking failure and increase the accuracy of re-detection. Comparative experiments with the state-of-the-art trackers demonstrate that the proposed method yields a robust and accurate tracking in complex tracking scenarios with interference factors including illumination variation, occlusion, out-of-view and background clutter.

## Figures and Tables

**Table 1:** Outline of the proposed tracking method.

**Input:** The target position $p_{1}$ and scale $s_{1}$ in frame 1;
**Output:** The target position $p_{t}$ and scale $s_{t}$ in frame *t*.
**Initialization:**
Crop out the image patch centered at $p_{1}$ and extract multi-category features $x_{1}$ in frame 1;
Initialize the filter $S_{f}$ using Equation (6) and set $r_{l}=1$ in frame 1;
Initialize the filter $S_{L}$ and $S_{s}$;
Initialize the SVM detectors h using Equation (9);
**for** t=2t=t+1,t⩽Num // Num is the number of frames in the video
Crop out the image patch centered at $p_{t-1}$ and extract multi-category features x in frame *t*;
// Translation estimation
Estimate the target position $p_{t}$ in frame *t* using Equation (8);
// Tracking failure detection
Compute the maximum response $A_{L}=\max S_{L}\left\{x\right\}$ of the filter $S_{L}$ at position $p_{t}$;
**if** $A_{L}=\max S_{L}\left\{x\right\}<T_{r}$
//Target re-detection
Activate detection module and return the candidate position $p_{tmp}$;
Compute the maximum response $A_{L_{\_tmp}}$ of the sample extracted at position $p_{tmp}$ on the filter $S_{L}$;
**if** $A_{L\_tmp}>T_{a}$
Rectify the target position $p_{t}=p_{tmp}$;
**elseif**
Maintain the target position $p_{t}$ and discard the candidate position $p_{tmp}$;
**end if**
**elseif**
The tracking failure doesn’t occur;
**end if**
**Output:** The target position $p_{t}$.
// Scale estimation
Construct scale pyramid centered at $p_{t}$ in frame *t* and estimate $s_{t}$ using filter $S_{s}$;
**Output:** The targe scale $s_{t}$ in frame *t*.
// Model update
Crop out the image patch centered at $p_{t}$ in frame *t* and extract multi-category features x;
**if** ttNu==0Nu==0
Update the feature tracking reliability $r_{l}$ using Equation (6); // Feature tracking reliability evaluation
Update the filter $S_{f}$ using Equation (7);
Update the scale filter $S_{s}$;
**end if**
**if** $A_{L}>T_{s}$
Update the long-term filter $S_{L}$ and update multiple SVM detectors h using Equation (10);
**end if**
**end for**

**Table 2:** Parameters of Experimental.

Name	Symbol	Value
Shape parameter of interpolation function	*a*	−0.75
Threshold of starting the detection module	$T_{r}$	0.15
Threshold of updating the Long-term filter	$T_{r}$	0.38
Threshold of adopting the re-detected result	$T_{a}$	1.5 $A_{L}$
Number of samples in sample space	$N_{s}$	50
Updating Period of tracker $S_{f}$	$N_{u}$	5
Regularization parameter in Equation (12)	λ	$2\times 10^{-7}$
Hyper-parameter of SVM detector	τ	1
